# Is deployement of diagnostic test alone enough? Comprehensive package of interventions to strengthen TB laboratory network: three years of experience in Burkina Faso

**DOI:** 10.1186/s12879-021-06012-y

**Published:** 2021-04-13

**Authors:** Riccardo Alagna, Adjima Combary, Elisa Tagliani, Léon Tinnoga Sawadogo, Tandaogo Saouadogo, Souba Diandé, Francis Ouedraogo, Daniela Maria Cirillo

**Affiliations:** 1 IRCCS San Raffaele Scientific Institute, Milan, Italy; 2 National Tuberculosis Program, Ouagadougou, Burkina Faso

**Keywords:** Tuberculosis, International cooperation, Technical assistance, Diagnostic

## Abstract

**Backgrounds:**

The laboratory plays a critical role in tuberculosis (TB) control by providing testing for diagnosis, treatment monitoring, and surveillance at each level of the health care system. Weak accessibility to TB diagnosric services still represents a big concern in many limited resources’ countries. Here we report the experience of Burkina Faso in implementing a comprehensive intervention packages to strengthen TB laboratory capacity and diagnostic accessibility.

**Methods:**

The intervention lasted from October 2016 to December 2018 and focused on two main areas: i) development of strategic documents and policies; ii) implementation of TB diagnostic technology.

National TB laboratory data were collected between 2016 and 2018 and evaluated according to five programmatic TB laboratory indicators: i) Percentage of notified new and relapse TB cases with bacteriological confirmation; ii) Percentage of notified new and relapse TB cases tested by Xpert MTB/RIF; iii) Percentage of notified, bacteriologically confirmed TB cases with a drug susceptibility testing (DST) result for rifampin; iv) Percentage of notified MDR-TB cases on the estimated number of MDR-TB cases; v) The ration between the number of smear microscopy and Xpert MTB/RIF tests. We compared these indicators between a 1 year (2016–2017) and 2 years (2016–2018) timeframe.

**Results:**

From 2016 to 2018, the percentage of bacteriologically confirmed cases increased from 67 to 71%. The percentage of new and relapse TB cases notified tested by Xpert MTB/RIF increased from 18% in 2016 to 46% in 2018 and the percentage of bacteriologically confirmed cases with an available DST result for rifampicin increased from 27% in 2016 to 66% in 2018.. The percentage of notified MDR-TB cases on the estimated number of MDR-TB cases in 2018 increased from 43% in 2016 to 78% in 2018. In 2018, the ratio between the number of smear microscopy and Xpert MTB/RIF tests decreased from 53% in 2016 to 21% in 2018.

**Conclusion:**

We demonstrated that the implementation of a comprehensive package of laboratory strengthening interventions led to a significant improvement of all indicators. External technical assistance played a key role in speeding up the TB laboratory system improvement process.

**Supplementary Information:**

The online version contains supplementary material available at 10.1186/s12879-021-06012-y.

## Background

The laboratory plays a critical role in tuberculosis (TB) control by providing testing for diagnosis, treatment monitoring, and surveillance at each level of the health care system. Despite representing an integral part of a continuum of care by providing evidence for decision making in clinical practice, diagnostic servicses are the weakest link in the cascade of care influencing only 60–70% of the healthcare decisions [1]. The diagnostic gap is a common thread of many infectious diseases, but it is much higher for TB [2].

Globally in 2019, there was a gap of about 2.9 millions between the number of incident and notified TB cases in the same year [3]. The missed cases were either not diagnosed or diagnosed but not reported. Despite the development of diagnostic tests able to provide rapid and accurate detection of TB and drug-resistant TB, poor accessibility to the health care system is still a big concern in many settings [4, 5]. In addition, on-field implementation studies have shown that diagnostic tests alone may fail to meet the expected impact on the TB care cascade if not implemented within the context of a strengthened health system [6].

Development of new TB diagnostic, treatment, and prevention tools has increased substantially over the last decades, leading to more than 20 new or updated World Health Organization (WHO) guidelines on different aspects of TB care [3, 7–9].

As a result of these rapid changes, National TB Programmes (NTP) have experienced difficulties in the prompt adoption and full implementation of TB laboratory policies and strategies [6, 10]. In this context, the WHO plays a crucial role in directing the international public health efforts through the coordination of technical assistance programmes to countries, partnerships, and initiativesaimed at facilitating the translation of policies into practice. The TB Supranational Reference Laboratory Network (SRLN) is a key WHO technical resource in supporting the strengthening of laboratory capacity in high TB burden countries [11].

The Emerging Bacterial Pathogens Unit (EBPU) of the Fondazione Centro San Raffaele part of San Raffaele Scientific Institute of Milan, Italy, was appointed as SRL and as WHO Collaborating Centre for TB laboratory strengthening (ITA-98) in 2006 and 2013, respectively. From 2016 to 2018, the EBPU-SRL Milan, thanks to the United States Agency for International Development (USAID) financial support, has served as a technical partner to the WHO Global TB Program to strengthen the TB diagnostic capacities and to support the Drug-Resistance Surveillance (DRS) activities in selected countries, including Burkina Faso..

The objective of the present study is to determine the impact of implementing a comprehensive package of interventionson the TB laboratory capacity and accessibility in Burkina Faso.

## Methods

### Setting

In 2016, the Burkina Faso TB three-tier laboratory network consisted of a National TB reference laboratory (NRL), 13 regional laboratories, and 97 peripheral laboratories. Both fluorescence and conventional light microscopy were the main TB diagnostic methods used. The Xpert MTB/RIF assay (Cepheid, Sunnyvale CA) was first introduced in the country in June 2013 with the placement of one 4-module GeneXpert machine (GX4) at the TB NRL. Subsequently, in October 2016, two additional GX4 instruments were installed at two regional laboratories. By November 2016, the GeneXpert network was expanded with additional 12 GX4 machines resulting in a total of 15 instruments covering all regions in the country.

Routine TB examination consisted on clinical evaluation, sputum smear microscopy, and chest radiography. Patients at high-risk of multidrug-resistant TB (MDR-TB), children, and people living with HIV (PLHIV) were tested by Xpert MTB/RIF, and patients with rifampin resistance results were referred to the NRL for genotypic testing for first-line anti-TB drugs.

### Intervention strategy

**Fig. 1:**
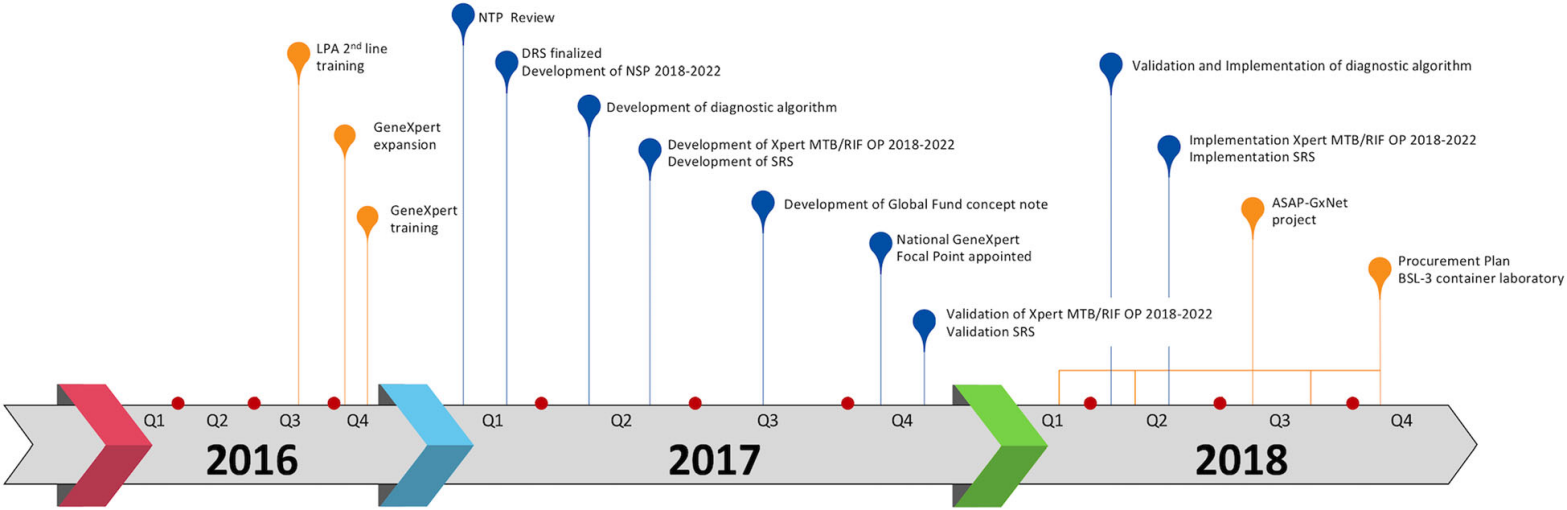
Timeline of TA activities carried out between October 2016 to December 2018**.** Orange dots represent the implmenetation of TB diagnostic guidelines and technology; Blue dots represent the development of strategic documents and policies. Line Probe Assay (LPA); National TB Programme (NTP); Drug-resistance Survey (DRS); Operational Plan (OP); Sample Referral Strategy (SRS): Biosafety Level-3 (BSL-3)

From October 2016 to December 2018, the EBPU-SRL Milan has provided extended and highly specialized technical assistance (TA) to the NTP and the national TB laboratory network of Burkina Faso. The TA focused on two main areas: i) development of strategic documents and policies; ii) implementation of TB diagnostic technologies (Fig. 1).

i) Development of strategic documents and policies
  The National TB Laboratory network assessment carried ot in 2017 led to the identification of the strengths and weaknesses of the overall system, while the DRS study provided information on national anti-TB drug resistance prevalence. Altogether, this information guided the NTP to determine the priority actions to be implemented and constituted the foundations for developing new or revising existing national policies and strategic plans such as the five-years National Strategic Plan (NSP) 2018–2022. Diagnostic algorithms had also been reviewed to move toward the END TB Strategy goal of universal access to rapid diagnostics and DST (Supplementary material: Figure S1, S2.1-S2.4). To this purpose, the NTP developed an Xpert MTB/RIF operational plan for the years 2018–2022 to strengthen the national GeneXpert network. Special attention had also been paid to the sample referral and transport system to enable the timely diagnosis of patients living in the countries remote areas.
  This comprehensive package of strategic and operational plans facilitated the development of the concept note for the Global Fund grant cycle 2018–2020 to ensure the proper financing of the TB laboratory priorities.
ii) Implementation of TB diagnostic technologies
  The development of strategic documents was interconnected with activities aimed at strengthening the TB national diagnostic services. By November 2016, the GeneXpert network was expanded with additional twelve 4-modules machines resulting in a total of 15 instruments covering all regions. A total of 60 laboratory technicians were trained to operate the GeneXpert machines, and more than 200 clinicians were instructed on TB patients referral and initiation of appropriate treatment based on Xpert results. To ensure the effective introduction of Xpert MTB/RIF test within the diagnostic routine, a pilot operational research project financed by the STOP TB Partnership named “Applying a Standardized Approach to Strengthen Performances of GeneXpert Networks” (ASAP-GxNet) was started [12]. The project aimed at strengthening the local managerial skills and at assessing in a standardised way the functionality of the GeneXpert network. Finally, the expansion of the GeneXpert network was accompanied by the strengthening of the diagnostic capacity at the NRL [13]. The activities included the implementation of Line Probe Assay (LPA) for second line drugs and the development of a procurement plan for a Biosafety Level-3 (BSL-3) container laboratory to be used for liquid culture and Drug-Susceptibility Testing (DST).

### Data sources and analysis

The NTP provided the TB case notification data from the national TB electronic database from 2016 to 2018. All data were anonymized and no sensitive patient information were used for data analysis. Data included TB notifications among new and previously treated patients stratified by type of diagnosis (i.e. clinical or bacteriological), test type (smear microscopy, Xpert MTB/RIF) and result. Access to data has been granted to the TB Surpanational Reference Laboratory of Milan by the National TB Programme of Burkina Faso within the framework of collaboration agreement signed between the two parties in 2011.

Quantitative data per year were imported to an Excel spreadsheet and analysed according to five programmatic TB laboratory indicators: i) Percentage of notified new and relapse TB cases with bacteriological confirmation; ii) Percentage of notified new and relapse TB cases tested with Xpert MTB/RIF assay; iii) Percentage of notified, bacteriologically confirmed TB cases with DST results for rifampin; iv) Percentage of notified MDR-TB cases on the estimated number of MDR-TB cases; v) Ratio between the number of smear microscopy and Xpert MTB/RIF tests.

TB notification data was used to calculate the five indicators and trends were compared over a 3 years time-frame.

## Results

To assess the impact of the development and implementation of TB diagnostic guidelines and the effectiveness of the TA provided by the SRL Milan, we monitored the following five indicators over a three-years timeframe, from 2016 to 20/20:

i) Notified new and relapse TB cases with bacteriological confirmation in 2016, 2017 and 2018

**Fig. 2:**
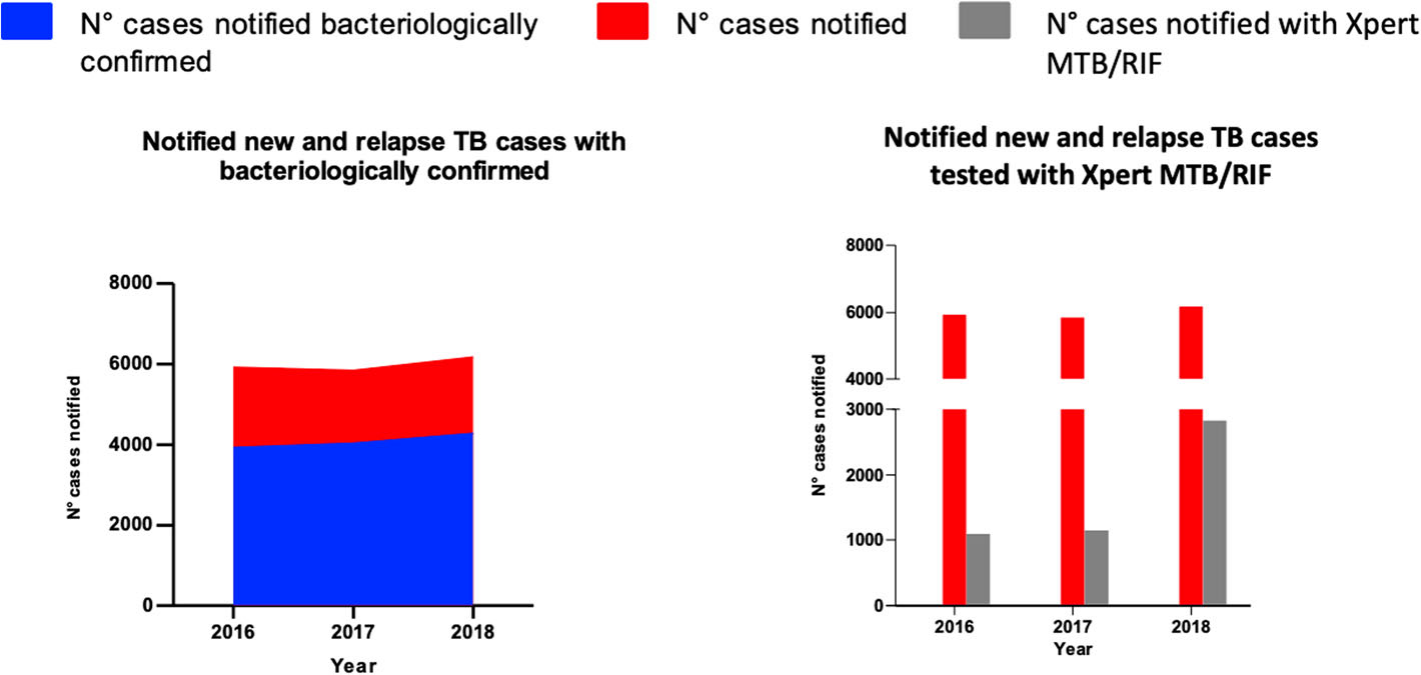
Laboratory indicators I and II. On the left, trend of number of new and relapse TB cases notified between 2016 and 2018 stratified based on type of diagnosis. On the right, the absolute number and percentage of new and relapse TB cases notified from 2016 to 2018 tested with Xpert MTB/RIF; in red notified cases clinically diagnosed, in blu notified cases Xpert MTB/RIF diagnosed

A total of 5918 and 5839 new and relapse TB cases were notified in 2016 and 2017, respectively. Of them, 3993 (67%) and 4044 (69%) were bacteriologically confirmed through smear microscopy and/or Xpert MTB/RIF (Fig. 2). In 2018, out of the 6166 new and relapse TB cases notified, 4288 (71%) were bacteriologically confirmed through smear microscopy and/or Xpert MTB/RIF (Fig. 2).

ii) Notified new and relapse TB cases tested with Xpert MTB/RIF in 2016, 2017 and 2018

The number of new and relapse TB cases notified tested with Xpert MTB/RIF remained stable in 2016 and 2017 with a totale of 1094/5918 (18%) and 1147/5839 (20%) in 2016 and 2017 respectively. Only in 2018, we observed a much higher increase in the number of notified TB cases that received an Xpert MTB/RIF test, up to 2826/6166 (46%) (Fig. 2).

iii) Notified, bacteriologically confirmed TB cases with DST result for rifampicin in 2016, 2017 and 2018

**Fig. 3:**
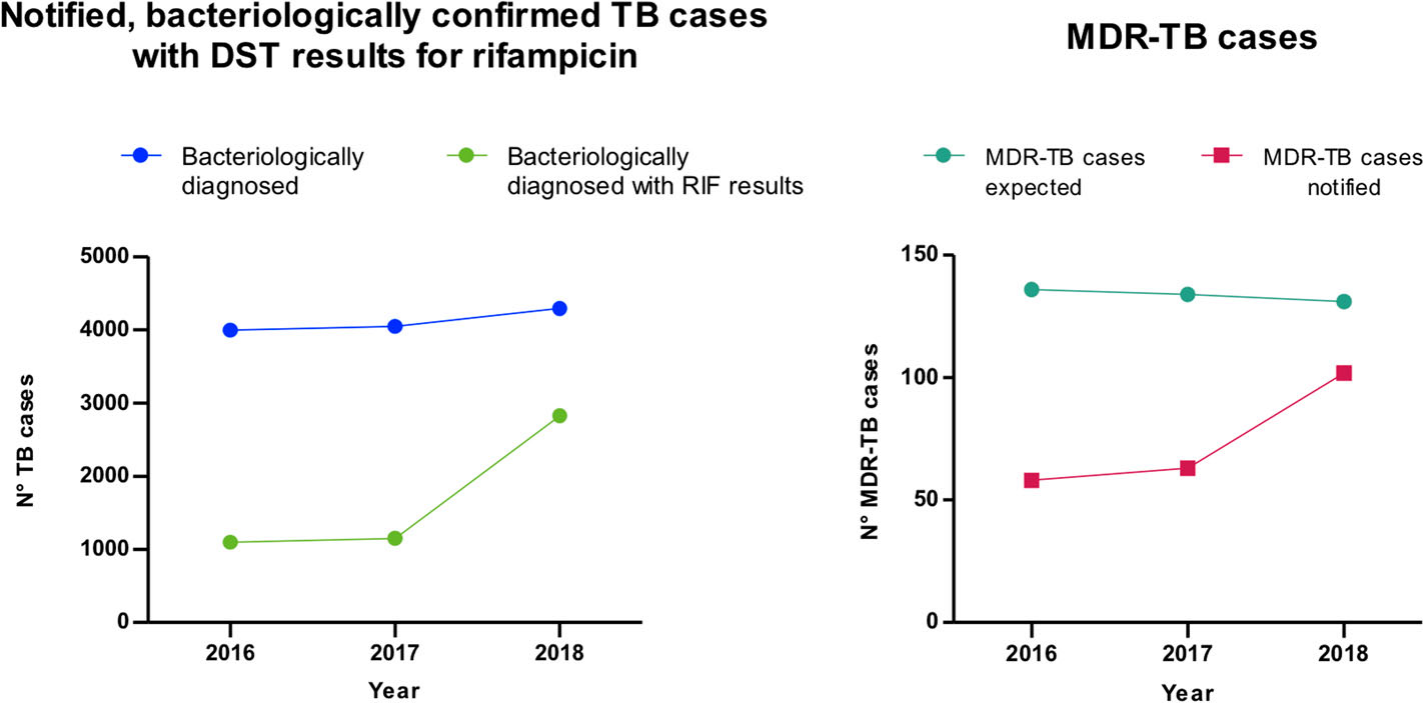
Laboratory indicators III and IV. On the left, number of notified bacteriologically confirmed TB cases compared to the number of bacteriologically confirmed TB cases with rifamicin result. On the right, number of MDR-TB cases notified over the number of estimatd MDR-TB cases

The number of bacteriologically confirmed TB cases with an available DST result for rifampicin increased from 27% in 2016 (1094/3993) to 28% in 2017 (1147/4044) (Fig. 3). Notably, the number of bacteriologically confirmed cases with an available DST result for rifampicin increased up to 66% (2826/4288) in 2018 (Fig. 3).

iv) Notified MDR-TB cases in 2016, 2017 and 2018

The number of notified MDR-TB cases (confirmed by Genotype MTBDR*plus* VER 2.0) over the number of estimated MDR-TB cases increased from 43% (58/136) in 2016 to 47% (63/134) in 2017. In 2018, we observed a much higher increase in the ratio between notified and estimated MDR-TB cases, upt to 78% (102/131).

xxii) Ratio between the number of smear microscopy and Xpert MTB/RIF tests

The utilization of Xpert MTB/RIF test in routine practice has been assessed by calculating the ratio between the number of smear-microscopy and Xpert MTB/RIF tests performed annually. This ratio decreased from 53% (58,510/1105) to 51% (61,755/1200) in 2016 and 2017 respectively. In 2018, the ratio between the number of smear microscopy and Xpert MTB/RIF test substantially decreased down to 21% (59,714/2826).

## Discussion

In this paper, we demonstrated that broader access to diagnostic services requires a comprehensive package of interventions that address multiple needs. This evidence is clearly supported by the improvement of all five indicators we monitored over the three-years timeframe. By the end of 2016, Burkina Faso experienced a 5-time increase in the number of 4-module GeneXpert instruments. This expansion was crucial to decentralize the diagnostic capacity of the country, but still not sufficient to drastically improve the evaluated indicators over a 1 year timeframe (2016–2017). We instead observed a strong improvement of the five indicators by the end of 2018, after the implementation of laboratory guidelines and strategies. Nonetheless, while all of evaluated indicators showed a substantial increase over time, the overall number of new and relapse TB cases that were bacteriologically confirmed showed a smaller change over the two-years timeframe (2016 to 2018). This can be explained by the fact that access to Xpert MTB/RIF testing was initially limited to smear positive patients at high-risk of MDR-TB, to avoid the rapid overload of the network and to promote a more gradual move toward universal DST.

Also the provision of technical assistance played an important role in speeding up the process of increasing diagnostic accessibility. This is shown by the change in the ratio between the number of smear microscopy and Xpert MTB/RIF tests performed. This indicator can be used to monitor how a country moves with regard to Xpert usage when compared to other settings [10, 14]. Between 2017 and 2018, Burkina Faso substantially increased the utilization of GeneXpert over microscopy, which resulted in a 30% decrease of the smear/Xpert ratio. Notably, this process was much faster as compared to other African countries with more experience on Xpert implementation such as Mozambique, Uganda, DR Congo, and Zimbabwe [10].

It is undoubtedly that interventions had a positive effect on the evaluated indicators and external assistance has played a fundamental role in speeding up the TB laboratory system improvement process. However, we acknowledge that additional programmatic factors, other than those evaluated in this work, such as community engagement to promote health seeking behaviours, improved TB communication strategies and support to TB advocacy groups, could have contributed to the improvement of the TB indicators.

## Conclusion

The experience of Burkina Faso shows that the country-wise implementation of laboratory guidelines is a requirement to rapidly advance the provision of TB laboratory services. We believe that, the lessons learnt over this three-years timeframe will be useful to other NTPs that aim to achieve concrete goals in strengthening their national TB laboratory networks. One of the key lessons learnt is the need for country commitment. These results could have not been achieved without a strong national commitment as this represents a sine qua non condition to ensure that the planned activities have a continuum on national TB control strategy. Another key lesson learnt is related to the need of building a roadmap of priority interventions guided by a comprehensive analysis that include the country’s epidemic situation, as well as the assessment of financial and, human resources and available network infrastructure. In ou specific context, timing played a crucial role, as Burkina Faso was able to achieve most of its results by conducting this comprehensive analysis and development of key operational plans the year before the Global Fund grant development. This was critical to secure the necessary funding to implement priority activities. Together with this clear activity plan, technical assistance played a key role in boosting the achievement of the obtained outcomes. Importantly, TA needs to be planned in advance with the country and must be provided over an adequate period of time rather than being delivered on-demand or through a single interaction. Supporting countries with a comprehensive package of technical assistance interventions that cover the entire donor’s grant cycle is preferable over a fragmented TA since this helps ensuring continuity while at the same time increasing the pace of change. Estimating TA financial needs is complex. Technical partners must work togehter with the NTP in identifying upfront the country needs for the implementation of the priority activities during the donor’s grant cycle. In this context, TA acts as an intermediary to best align and leverage donor’s and recipient’s needs. Security of funds for external technical assistance has multiple benefits as it allows to work with the same TA provider over a prolonged period of time and, therefore, to build a strong relationship between the partners and facilitate the systematic transfer of skills. Besides the TA plan and secured funding, also the consultant individual capacity and skills in assisting the country is a key factor, as it should promote the establishment of an environment fostering the autonomous development of the country staff rather than a dependency culture.

## Recommendations

The experience from Burkina Faso can help outlining some recommendations which may inform countries with similar challenges on future technical assistance interventions to strengthen laboratory system:

- Conduct a comprehensive laboratory network assessment to identify TB laboratory needs;
- Identify a package of actions that are feasible and acceptable to both those receiving and those delivering supports;
- Ensure that TB laboratory needs are translated into the National Strategic Plan;
- Update the national diagnostic algorithms to ensure optimal utilization of laboratory resources;
- Develop a national sample referral system to improve accessibility of laboratory services;
- Develop an Xpert Operational Plan based ASAP-GxNet assessment tools;
- Ensure that policies are effectively implemented by health professional capacity building;
- Ensure country ownership by enhancing country’s capacity to determine its own development and decide its priority activities. Ownership also leads to high-level intention to maintain and achieve results.
- Ensure engagement of an external consultant that fits with the demands of health development in low-resource settings and has the ability to enhance an autonomous development environment rather than a dependency culture;
- Plan external technical assistance upfront and ensure its provision over a prolonged period rather than a request that is answered immediately or through a single interaction;
- Ensure robust funding mechanism to support external technical assistance providers as this allows external providers to appropriately shape the activities according to the country needs and to attend the expected benefit.

## Supplementary Information

**Additional file 1.**

## Data Availability

The datasets generated and/or analysed during the current study are not publicly available due to data protection requirements but are available from the corresponding author on reasonable request.

## Abbreviations

BSL-3: Biosafety Level-3
DRS: Drug-Resistance Surveillance
DST: Drug-susceptibility testing
EBPU: Emerging Bacterial Pathogens Unit
GX4: GeneXpert machine 4-module
LPA: Line Probe Assay
MTB: *M. tuberculosis*
MDR-TB: Multidrug-resistant TB
NRL: National TB reference laboratory
NSP: National Strategic Plan
NTP: National TB Program
OP: Operational Plan
PLHIV: People living with HIV
RIF: Rifampicin
SRLN: TB Supranational Reference Laboratory Network
SRS: Sample Referral Strategy
TA: Technical assistance
TB: Tuberculosis
USAID: United States Agency for International Development
WHO: World Health Organization
