# Supplementary material for: Is deployement of diagnostic test alone enough? Comprehensive package of interventions to strengthen TB laboratory network: three years of experience in Burkina Faso
Source: BMC Infect Dis. 2021 Apr 13;21:346. doi: 10.1186/s12879-021-06012-y (PMC8042973; doi:10.1186/s12879-021-06012-y)
Supplement: Supplementary file 1 — Additional file 1. [file 12879_2021_6012_MOESM1_ESM.docx]

Supplementary material: Algorithm


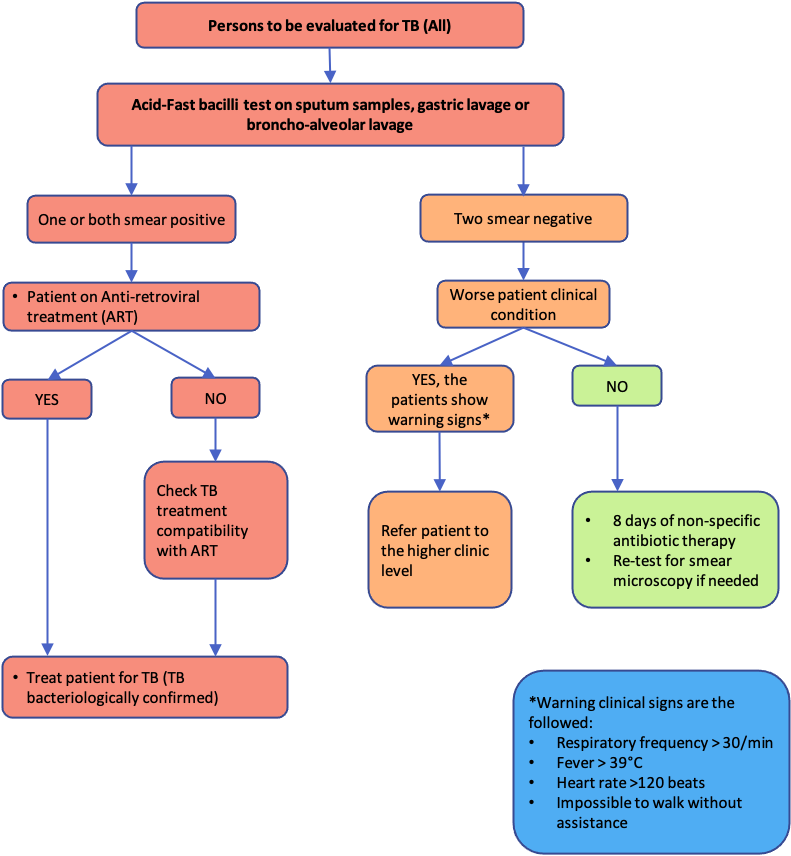


Figure S1: National TB Programme’s algorithm available in 2016


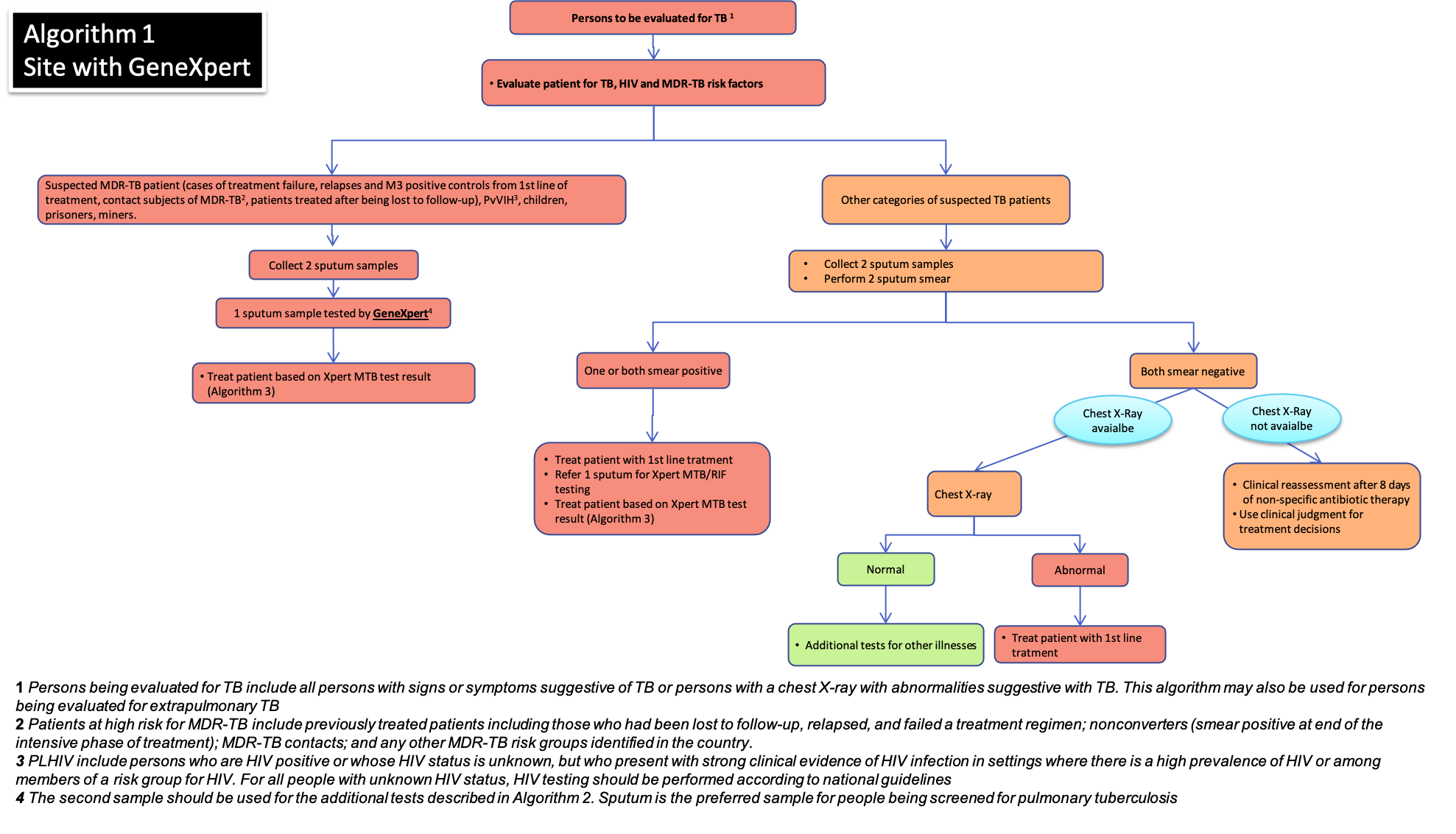


Figure S2.1: National TB Programme’s algorithms developed and implemented in 2017 – GeneXpert site algorithm


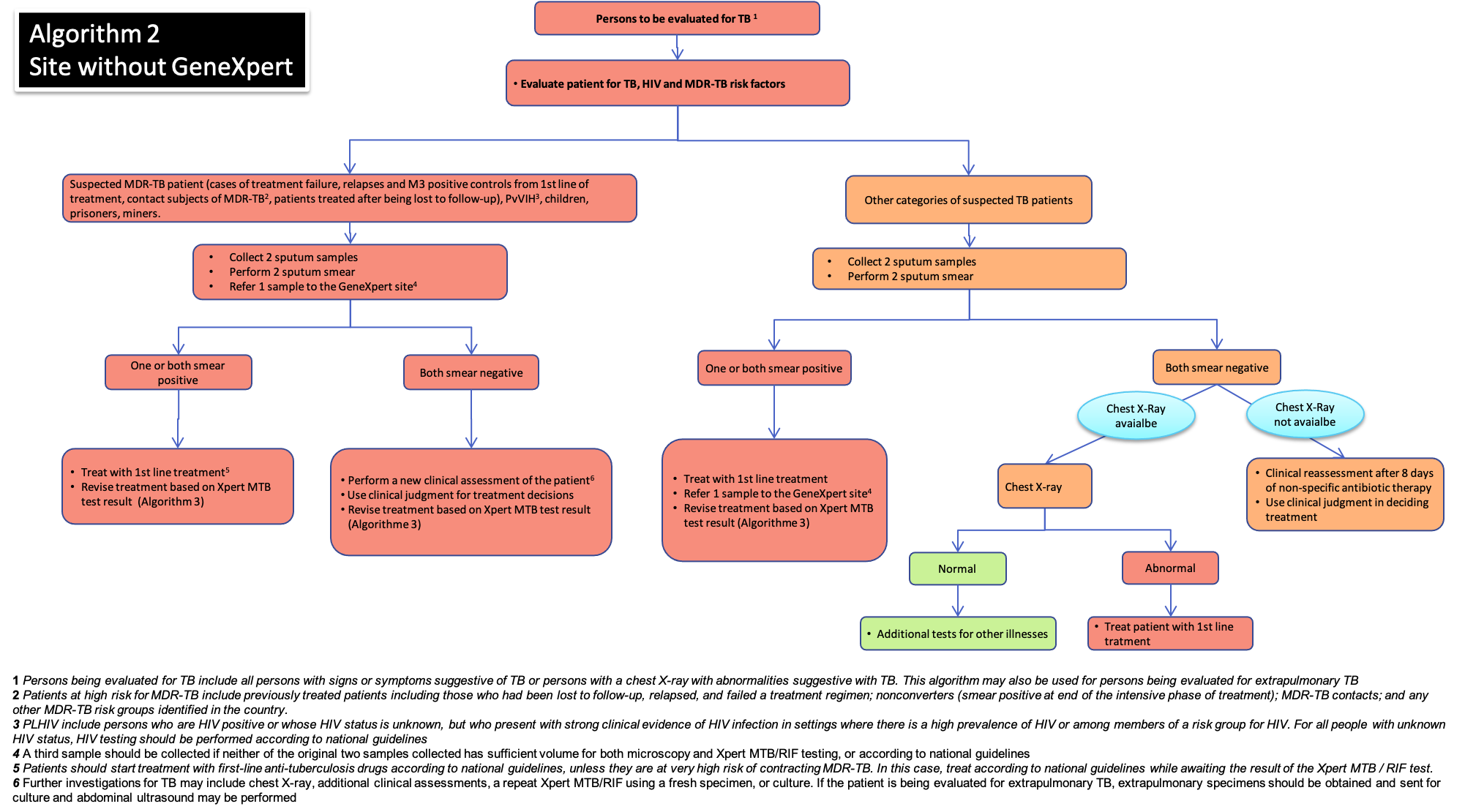


Figure S2.2: National TB Programme’s algorithms developed and implemented in 2017 – Non GeneXpert site algorithm


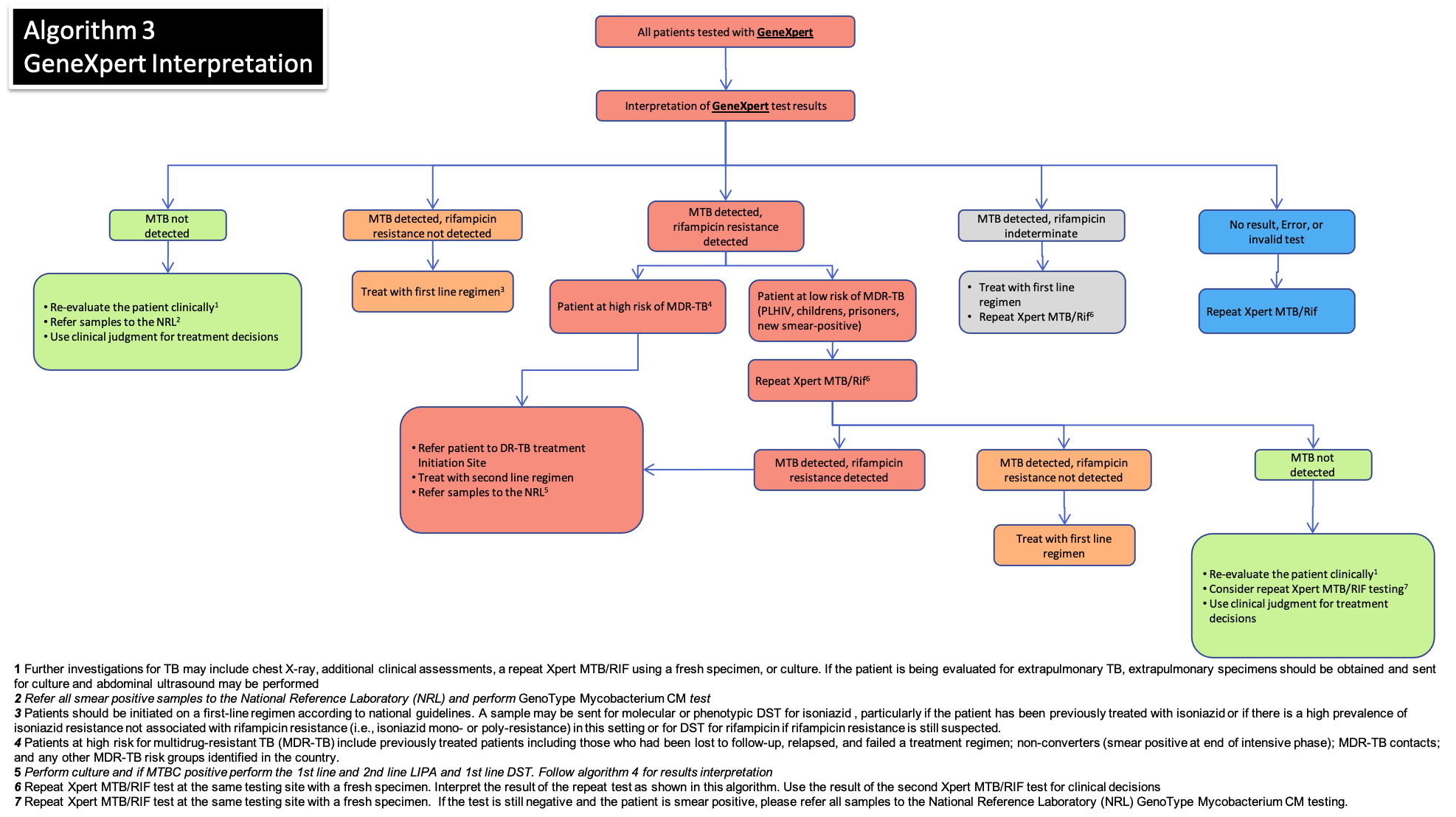


Figure S2.3: National TB Programme’s algorithms developed and implemented in 2017 – GeneXpert interpretation algorithm


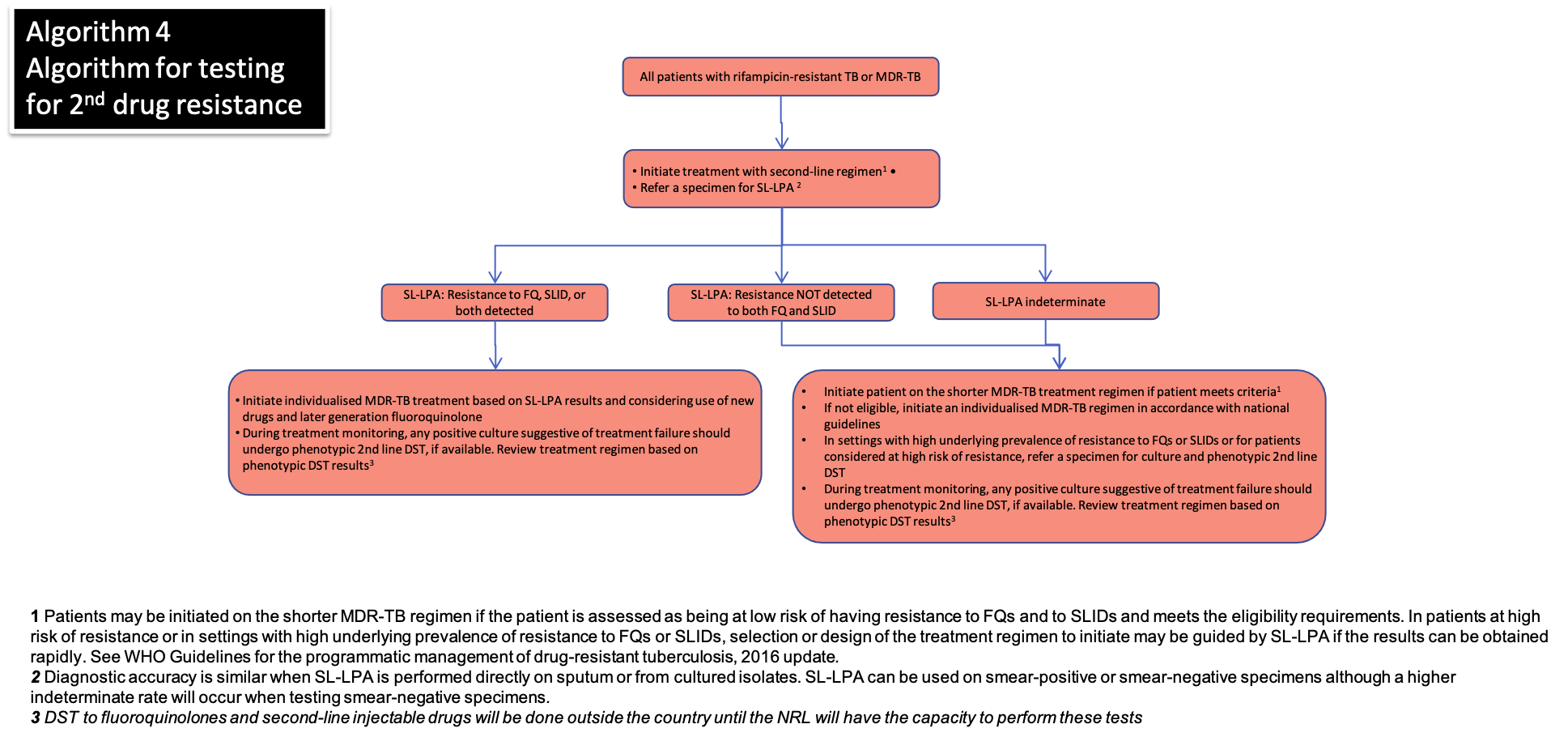


Figure S2.4: National TB Programme’s algorithms developed and implemented in 2017 – TB-MR, pre-XDR-TB and XDR-TB diagnostic algorithm
